# Amyloid Fibrils of Pea Protein Isolated Induced by Cold Plasma Treatment and Its Stabilization on High Internal Phase Emulsions

**DOI:** 10.1002/fsn3.71038

**Published:** 2025-09-29

**Authors:** Jing Wang, Jun‐Xiang Liu, Xiu‐Bin Liu, Najla AlMasoud, Abderrahmane Aït‐Kaddour, Rana Muhammad Aadil, Zhi‐Wei Liu

**Affiliations:** ^1^ Hunan Food and Drug Vocational College Changsha China; ^2^ College of Food Science and Technology Hunan Agricultural University Changsha China; ^3^ Department of Chemistry, College of Science Princess Nourah Bint Abdulrahman University Riyadh Saudi Arabia; ^4^ Université Clermont Auvergne, INRAE, VetAgro Sup UMRF Aurillac France; ^5^ Department of Food Technology, Faculty of Agroindustrial Technology University of Padjadjaran Sumedang Jawa Barat Indonesia; ^6^ National Institute of Food Science and Technology University of Agriculture Faisalabad Pakistan; ^7^ Guangdong Key Laboratory of Food Intelligent Manufacturing Foshan University Foshan China; ^8^ Changsha Innovation Institute for Food Changsha China

**Keywords:** amyloid fibrils, cold plasma, high internal phase emulsions, pea protein isolates

## Abstract

The potential of forming pea protein isolate (PPI) amyloid fibrils promoted by cold plasma (CP) treatment (40 kV; 0, 3, 5, and 7 min), heat treatment (85°C for 12 h), and its ability to stabilize high internal phase emulsions (HIPEs) was investigated. Results indicated that rod‐like amyloid fibrils of PPI were successfully formed and promoted by CP treatment, with a 12 nm diameter and 132–261 nm average length, as confirmed by Thioflavin T (ThT) fluorescence spectroscopy, SEM, and TEM. The globular structure unfolding, cleavage of the backbone of PPI by CP treatment, release of the building block “β‐strands” structure, and assembly of the building blocks into fibrils during the fibrillation process were confirmed by SDS‐PAGE, FTIR, fluorescence spectroscopy, and hydrophobic analysis. Compared with native PPI, fibrous PPI exhibited a strong capacity for stabilizing HIPEs, and the ability to stabilize HIPEs was PPI fibrils length dependent. Compared with HIPEs stabilized by long fibrils (CP5PF‐8 and CP5PF‐12), the HIPEs prepared by short fibrils (CP5PF‐2 and CP5PF‐4) displayed better thermal stability. Moreover, CP5PF‐stabilized HIPEs exhibited excellent ionic and storage stability, while PPI amyloid fibrils with shorter lengths (CP5PF‐2 and CP5PF‐4) showed superior performance compared with longer fibrils lengths (CP5PF‐8 and CP5PF‐12).

## Graphical abstract

Rod‐like amyloid fibrils of PPI were successfully formed and promoted by CP treatment. Fibrous PPI exhibited a strong capacity for stabilizing HIPEs which is fibrils length dependent. Short fibrils displayed better thermal stability, ionic, and storage stability.

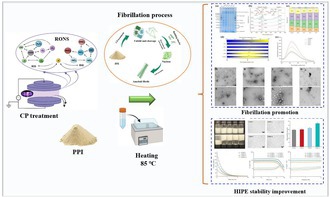

## 1 Introduction

Protein is commonly used as emulsifiers or foaming agents due to its excellent functional properties and high nutritional value (Akharume et al. 2021; Pazla et al. 2023; Othman et al. 2024). Plant protein foods are gaining increasing research attention globally due to their health and environmental benefits compared to traditional animal protein foods (Bi et al. 2022). Among various plant proteins, pea protein isolate (PPI) is one of the most potential alternatives due to its high yield, excellent nutritional value, high bioavailability, and hypoallergenicity (Kornet et al. 2022). Normally, the functional properties of proteins determine their applicability across a wide range of food‐related applications; for instance, the interfacial properties dictate their functionality as emulsifiers or foaming agents (Xu, Ma, et al. 2023). However, the diverse composition, compact native structure, and low solubility of plant proteins often pose challenges to stable adsorption at the oil–water interface (Zhang et al. 2023). Therefore, structural modification of plant proteins, especially PPI, to improve their emulsion properties is important to broaden their applications in the food industry.

Food protein amyloids fibrils are typical unbranched protein fibrils approximately 10 nm in diameter and exceeding 1 μm in contour length. These fibrils are constructed by β‐strands oriented perpendicular to the fibril axis, forming a repetitive and stacked cross‐β structure (Xu, Zhou, et al. 2023). Numerous studies have demonstrated that food proteins, such as whey protein (α‐lactalbumin, β‐lactoglobulin), egg white protein (lysozyme, ovalbumin), soy protein, and pea protein, exhibit significant potential for in vitro fabrication into amyloid fibrils under acidic heat conditions (pH = 2, 80°C–90°C) (Zhang et al. 2024). Due to the high stiffness, high aspect ratio, and highly ordered structure, fibrillar food protein is recognized as a promising approach for promoting and modulating the beneficial functions of proteins (Xu, Ma, et al. 2023). Commonly, the degree and efficiency of protein conversion to amyloid fibrils are directly relevant to the protein native structure. Due to more compact structure and lower solubility of plant proteins, the key step of backbone hydrolysis and release of building blocks (β‐strands) for amyloid fibril formation is hindered, resulting in prolonged fibrillation times (Li et al. 2023; Halavach 2024). To address these challenges, sorts of strategies have been exploited, including nuclei induction (Yang, Song, et al. 2024), enzymatic (Pi et al. 2023), and physical pretreatment (Afkhami et al. 2023; Chen et al. 2024; Liu, Yang, et al. 2024b). Among these, physical treatments have garnered extreme attention due to their environmentally friendly and high efficiency. Tong et al. (2022) reported that ultrasound pretreatment results in exposing the hydrophobic regions of soybean protein isolate, thereby promoting its hydrolysis and amyloid fibril formation. Analogously, Liu et al. (2024a) found that pea protein pretreated with a low‐frequency magnetic field accelerated amyloid fibril formation, due to preunfolding its conformational structure. In recent years, cold plasma (CP), a nonthermal and sustainable food processing technique, has gained extensive interest for protein modification. Previous studies confirmed CP disturbs protein conformation, unfolding its globular structure and cleaving the polypeptide backbone, thereby mimicking enzymatic hydrolysis (Liu et al. 2022). These features make CP a promising approach for promoting protein amyloid fibril formation. Our prior research demonstrated that CP treatment combined solely with heat treatment significantly enhanced the fabrication of ovalbumin amyloid fibrils (Liu et al. 2025). Although CP has been demonstrated to improve the fibrillation kinetics of protein by efficiently reducing the time of the lag phase, the related functional properties of CP‐induced amyloid fibrils of protein have not been systematically investigated, particularly in stabilizing high internal phase emulsions.

Therefore, the objective of the current study was to (1) evaluate the formation of PPI amyloid fibrils facilitated by CP treatment in combination with heat treatment and (2) investigate the capacity of CP‐induced PPI amyloid fibrils in stabilizing HIPEs. To achieve these objectives, the self‐assembly characteristics and amyloid fibril formation of PPI during fibrillation incubation were monitored using ThT fluorescence spectroscopy, dynamic light scattering (DLS), scanning electron microscopy (SEM), and transmission electron microscopy (TEM) analysis. Structural changes in PPI were analyzed using sodium dodecyl sulfate polyacrylamide gel electrophoresis (SDS‐PAGE), Fourier transform infrared spectroscopy (FTIR), intrinsic fluorescence spectroscopy, and hydrophobicity analysis. The ability of CP‐induced PPI amyloid fibrils to stabilize HIPEs was evaluated using optical microscopy, confocal laser scanning microscopy (CLSM), and rheological analysis. Additionally, the thermal, ionic, and storage stability of HIPEs were further conducted.

## 2 Material and Methods

### 2.1 Materials and Chemicals

Pea protein isolated with a purity of 98% was purchased from Shanghai Yingxin Laboratory Equipment Co. Ltd. (Shanghai, China). ThT was provided by Sigma‐Aldrich Co. Ltd. (St. Louis, MO, USA). Protein markers were provided by SMOBiO Technology Inc. (Taiwan). All other chemicals were reagent‐grade.

### 2.2 CP Treatment

The CP treatment was conducted using a dielectric barrier discharge (DBD) plasma generator (Nanjing Sumant Electronics Co. Ltd., China). The detailed procedure for plasma treatment of PPI samples followed our previous studies (Liu et al. 2025). PPI (10 mg/mL) was dispersed in deionized water and stirred with a magnetic stirrer for 12 h to ensure sufficient hydration. The PPI sample solution (5 mL) was loaded into a quartz dish. CP treatment was conducted at 40 kV with a frequency of 14 kHz. The treatment times were set to 0, 3, 5, and 7 min, respectively. PPI samples treated with CP for 0, 3, 5, and 7 min were designated as CP‐0, CP‐3, CP‐5, and CP‐7, respectively. Native PPI, which is the sample without CP or thermal treatment, was designated as the control. After treatment, the sample was used for PPI amyloid fibril preparation.

### 2.3 Preparation of PPI Amyloid Fibrils

The preparation of PPI amyloid fibrils followed our previous studies, with slight modification (Liu et al. 2025). The CP‐treated PPI samples were heated in a water bath at 85°C with stirring at 100 rpm for 12 h. The samples were collected at various time points (0, 0.5, 1, 2, 4, 6, 8, 10, and 12 h). They were cooled in ice water immediately and stored at 4°C for further analysis. The amyloid fibrils prepared from CP‐treated PPI for 5 min and fibrillated for 2, 4, 8, and 12 h were labeled as CP5PF‐2, CP5PF‐4, CP5PF‐8, and CP5PF‐12, respectively.

### 2.4 ThT Fluorescent Analysis

ThT fluorescence was used to monitor the formation of PPI amyloid fibrils (Tang et al. 2024). ThT was dissolved in phosphate buffer (10 mM phosphate, 150 mM NaCl, pH 7.0) to prepare a 1 mM ThT stock solution, which was wrapped with aluminum foil and stored at 4°C. A 50 μM working solution was obtained by diluting the stock solution with phosphate buffer. Forty microliters of the sample (10 mg/mL) were added to 4 mL of the ThT working solution. It was incubated at a temperature of 25°C for 20 min. A microplate spectrophotometer (Varioskan Flash, Thermo Fisher Scientific, Finland) was employed to measure the fluorescence intensity of samples with an excitation wavelength of 440 nm and an emission wavelength of 480 nm. The excitation and emission slits were set to 12 nm.

### 2.5 TEM

The PPI amyloid fibrils sample was diluted to 0.1 mg/mL with deionized water. The sample solution was then dripped on a copper grid and negatively stained with 2% uranyl acetate. The microstructure of the PPI amyloid fibril was captured using a TEM (JEM1200ex; JEOL Ltd., Tokyo Metropolitan, Japan) operated at 100 keV. Fiji ImageJ software was used for further image analysis.

### 2.6 Sem

The PPI amyloid fibrils samples were freeze‐dried. The samples were fixed to a copper stub using double‐sided tape and coated with gold to ensure conductivity, and observed using an SEM (JSM‐6380LV; Hitachi, Tokyo, Japan) at an accelerating voltage of 25 kV.

### 2.7 SDS‐Page

Electrophoresis was performed using a WIX‐Multipro2 electrophoresis system (Wix Technology Co. Ltd., Beijing, China) as followed by our previous study. Sample (1 mg/mL, 0.1 M phosphate buffer solution (PBS) pH 7.0) and reducing buffer (4:1, *v/v*) were combined and heated to 90°C for 10 min. Acrylamide gels (5% stacking gel, 10% separating gel) were subjected to electrophoresis using an electrophoresis apparatus operating at 75 V for a period of 3 h. An aliquot of 10 μL samples and 5 μL marker were loaded. Following electrophoresis, the gel was stained for 1 h and then destained for 3 h (Liu et al. 2021).

### 2.8 FTIR Measurement

FTIR spectra of PPI samples were recorded using an IRAffinity‐1 spectrometer (Shimadzu, Japan) (Liu et al. 2025). Lyophilized samples (30 mg) were finely ground and mixed with 3.0 g of dried potassium bromide (1.0% w/w). The mixture was compressed into transparent tablets for analysis. The spectral scanning range was from 400 to 4000 cm^−1^ with a resolution of 4 cm^−1^.

### 2.9 Intrinsic Fluorescence Analysis

The change in intrinsic fluorescent intensity of PPI samples during the fibrillation incubation was monitored by fluorescence spectrophotometer (F‐7000; Hitachi, Japan) (Liu et al. 2025). The diluted samples (1 mg/mL) were scanned with a speed of 200 nm/min, an emission range of 300–400 nm, an excitation wavelength of 280 nm, and excitation/emission slit widths of 2.5 and 5 nm.

### 2.10 Surface Hydrophobicity Determination

Surface hydrophobicity of PPI samples during fibrillation incubation was determined using the ANS probe (Liu et al. 2025). An aliquot of 8 μL of ANS solution (5 mM in 0.1 M phosphate buffer, pH 7.0) was mixed with 1 mL of the PPI sample (1 mg/mL). The mixture was incubated in the dark for 30 min. Fluorescence intensity was recorded using a fluorescence spectrophotometer with the parameter: 380 nm in excitation wavelength with a slit of 2.5 nm; 400–600 nm in emission wavelength with a slit of 5 nm.

### 2.11 Particle Diameter and Size of Oil Droplets Measurement

The particle size distribution of PPI samples (1 mg/mL, diluted with deionized water) was measured using a Zetasizer Nano ZS system (Malvern Panalytical, UK) (Liu et al. 2025). The refractive index of the protein was set to 1.45, and the refractive index of the continuous phase was set to 1.33. The size of oil droplets in HIPEs was determined using a laser particle size analyzer (Mastersizer 3000; Malvern Panalytical, UK) with the following parameter settings: oil refractive index = 1.47, distilled water refractive index = 1.33, and 10% obscuration.

### 2.12 HIPEs Preparation

PPI amyloid fibrils fabricated from CP‐treated PPI for 5 min with different fibrillation times (2, 4, 8, and 12 h) were used as the continuous phase. The continuous phase (10 mg/mL) was mixed with the oil phase (soybean oil) at a volume ratio of 1:3 (*φ* = 0.75) and homogenized at 12,000 rpm for 2 min (Liu et al. 2025).

### 2.13 Optical Microscopy Analysis

An aliquot of 50 μL of the emulsion (mixed 1:1 with 0.1% SDS) was placed on a glass slide and covered with a coverslip. The glass slide was transferred to a microscope (Olympus CX31 microscope, Tokyo, Japan) and observed under a 10× eyepiece and a 10× objective lens. The pictures of the oil droplets were captured using the microscope's built‐in camera (Liu et al. 2025).

### 2.14 CLSM

The microstructure of the oil droplets in the HIPEs was further observed using a CLSM600 confocal laser scanning microscope (Ningbo Sunlight, China). The PPI samples were stained with Fast Green (0.5% ethanol solution), while the oil phase was stained with Nile Red (0.5% ethanol solution). The fluorescent probes Fast Green and Nile Red were excited at 625 and 488 nm, respectively. Fluorescent images of the HIPE samples were captured at a magnification of 40×.

### 2.15 Rheological Analysis of HIPEs

The rheological properties of the HIPEs were analyzed using a rotational rheometer equipped with a 40 mm flat plate (Malvern Instruments Ltd.) (Liu et al. 2025). The gap between the plates was maintained at 1.0 mm. In flow sweep mode, the apparent viscosity of the emulsion was measured at shear rates ranging from 0.1 to 100 s^−1^. In dynamic strain mode, the storage modulus (G′) and loss modulus (G″) of the HIPEs were measured at a frequency of 1.0 Hz and a strain ranging from 0.01% to 100%. In frequency sweep mode, the G′ and G″ of the emulsion were measured at a constant strain of 0.03% over a frequency range of 0.1 to 10 Hz.

### 2.16 Thermal Stability of HIPEs

An aliquot of 5 mL of freshly prepared HIPEs was transferred to glass bottles with lids and heated in a water bath at different temperatures (60°C, 75°C, and 90°C) for 60 min. The samples were then cooled to room temperature in an ice water bath. The appearance of the HIPEs was visually inspected and documented, and the droplet size and distribution were analyzed using an optical microscope. Rheological properties were also characterized, and photographs were collected for record purposes.

### 2.17 Ionic Stability of HIPEs

An aliquot of 5 M NaCl stock solution was added to the dispersed phase to achieve final concentrations of 100, 300, and 500 mM. The aqueous and oil phases were subsequently mixed and homogenized as described previously. The appearance of the HIPEs was visually inspected and documented, while the droplet size and distribution were analyzed using an optical microscope. Rheological properties were also characterized and recorded.

### 2.18 Storage Stability of HIPEs

An aliquot of 5 mL HIPEs was placed in 10 mL glass bottles and stored at 4°C for 7, 14, and 28 days, respectively, with the bottles inverted. The appearance of the emulsion was visually observed and recorded for each storage cycle, and then the droplet size and distribution of the HIPEs were observed using an optical microscope, and the rheological properties were characterized. Photographs were collected.

### 2.19 Statistical Analysis

All experimental data were obtained by measuring three times in parallel. Statistical analysis was performed using Excel 2010 and SPSS 25.0, and the results are expressed as mean ± standard deviation. The significance level was set at *p* < 0.05. Data analysis and graph plotting were performed using Origin 2019.

## 3 Results and Discussion

### 3.1 Amyloid Fibrils of PPI Induced by CP Treatment

#### 3.1.1 ThT Fluorescent

**FIGURE 1:**
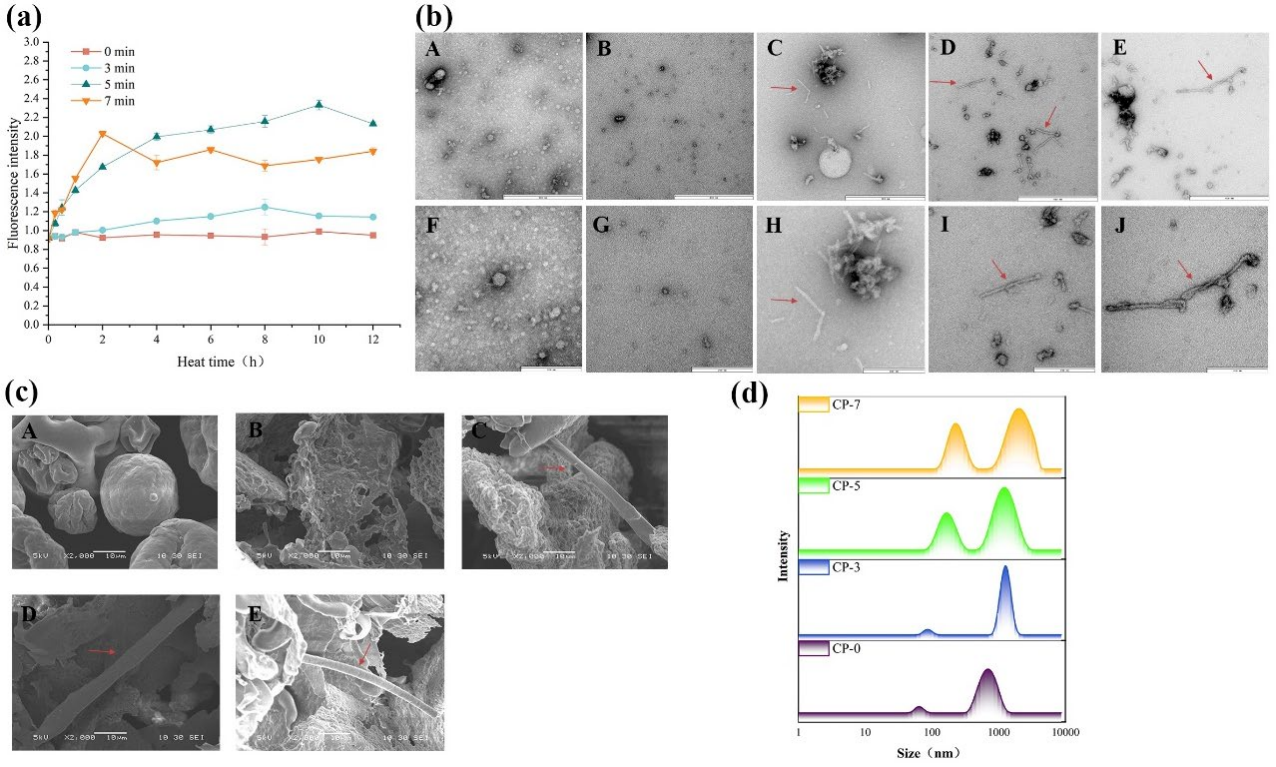
(a) Changes in ThT fluorescence intensity of PPI with different CP treatment times during the fibrillation process. (b) TEM analysis of PPI amyloid fibrils. (A, F) were native PPI; (B, G), (C, H), (D, I), (E, J) were PPI amyloid fibrils prepared by CP‐treated PPI for 0, 3, 5, and 7 min, respectively, with a fibrillation time of 10 h. The scale bar of A, B, C, D, and E is 600 nm, and the scale bar of F, G, H, I, and J is 200 nm. (c) SEM analysis of PPI amyloid fibrils; A is the natural PPI, and B, C, D, and E were PPI amyloid fibrils prepared by CP‐treated PPI for 0, 3, 5, and 7 min, respectively, with a fibrillation time of 10 h. The scale bar is 10 μm. (d) The particle size distribution of PPI amyloid fibrils prepared by CP‐treated PPI for 0, 3, 5, and 7 min, respectively, with a fibrillation time of 10 h. PPI samples treated with CP for 0, 3, 5, and 7 min were designated as CP‐0, CP‐3, CP‐5, and CP‐7, respectively.

ThT fluorescence analysis is widely regarded as a “gold standard” method for monitoring the formation of protein amyloid fibrils during the fibrillation process due to the dye's high efficiency and sensitivity in binding to protein amyloid fibrils (Bolder et al. 2007). The change in ThT fluorescence intensity of the CP‐treated PPI samples with 0, 3, 5, and 7 min during a 12 h fibrillation process was depicted in Figure 1a. Compared with CP‐0, the ThT fluorescence intensity of the CP‐5 and CP‐7 increased rapidly in the initial 2 h incubation time, reaching its maximum for CP‐7. With prolonged heating, the ThT fluorescence intensity of CP‐3 gradually increased, peaking at 8 h and 10 h, respectively, while the ThT value for CP‐7 decreased steadily. These results indicated that the amyloid fibril structures were successfully formed and promoted by CP treatment in combination with heat treatment (Yu et al. 2024). This phenomenon aligns with previous studies, which reported that ovalbumin amyloid fibrils were effectively facilitated by CP treatment alone (Liu et al. 2025). The decrease in ThT fluorescence intensity for CP‐7 after 2 h fibrillation time might be due to the fracture of the mature fibrils of the PPI fibrils shielding the ThT binding sites on their surface (Liu, Yang, et al. 2024a). Similar findings were reported that the ThT fluorescence intensity of soy protein (Wan and Guo 2019) and cowpea protein amyloid‐like fibrils (Li et al. 2021) was significantly reduced after prolonged heating time. Among all treatments, CP‐5 exhibited the highest fibril assembly capacity, suggesting that moderate CP‐mediated oxidation was superior to protein backbone cleavage and structural unfolding, thus facilitating protein amyloid fibrils formation (Liu, Chen, et al. 2024; Liu, Tang, et al. 2024).

#### 3.1.2 Amyloid Fibril Morphology

To confirm the consequence of ThT fluorescent analysis, TEM and SEM analyses were employed to visualize the formation and morphology of PPI amyloid fibrils. The TEM results for native and CP‐treated PPI samples with 0, 3, 5, and 7 min after 10 h of fibrillation were presented in Figure 1b and the length of fibrils was measured by ImageJ. Native PPI exhibited spherical aggregates with an average diameter of 26.94 nm. After 10 h of fibrillation, CP‐0 retained its spherical aggregates with fewer diameters, whereas short rod‐like fibrous structures were observed for CP‐3, CP‐5, and CP‐7. Amyloid fibrils from CP‐3, CP‐5, and CP‐7 exhibited a similar diameter of approximately 12 nm, with average lengths of 132, 217, and 261 nm, respectively. The results indicated that the length of amyloid fibrils increased with prolonged CP treatment time. SEM analysis (Figure 1c) supported these findings, showing regular spherical structures in native PPI and lamellar structures in CP‐0. In contrast, rod‐like amyloid fibril structures with smooth surfaces were observed in samples treated with CP for 3, 5, and 7 min. Analogously, Feng et al. (2024) reported that smooth rod‐like structures of gluten amyloid fibrils were observed by SEM, which formed from enzymatic hydrolysis peptides of gluten at a temperature of 37°C for 36 h. These observations were consistent with the size distribution analysis (Figure 1d), where particle size distribution peaks shifted to larger sizes as CP treatment time increased, indicating that fibrillation of PPI was successfully promoted by CP treatment.

#### 3.1.3 Structure Characteristic of PPI During Fibrillation Process

**FIGURE 2:**
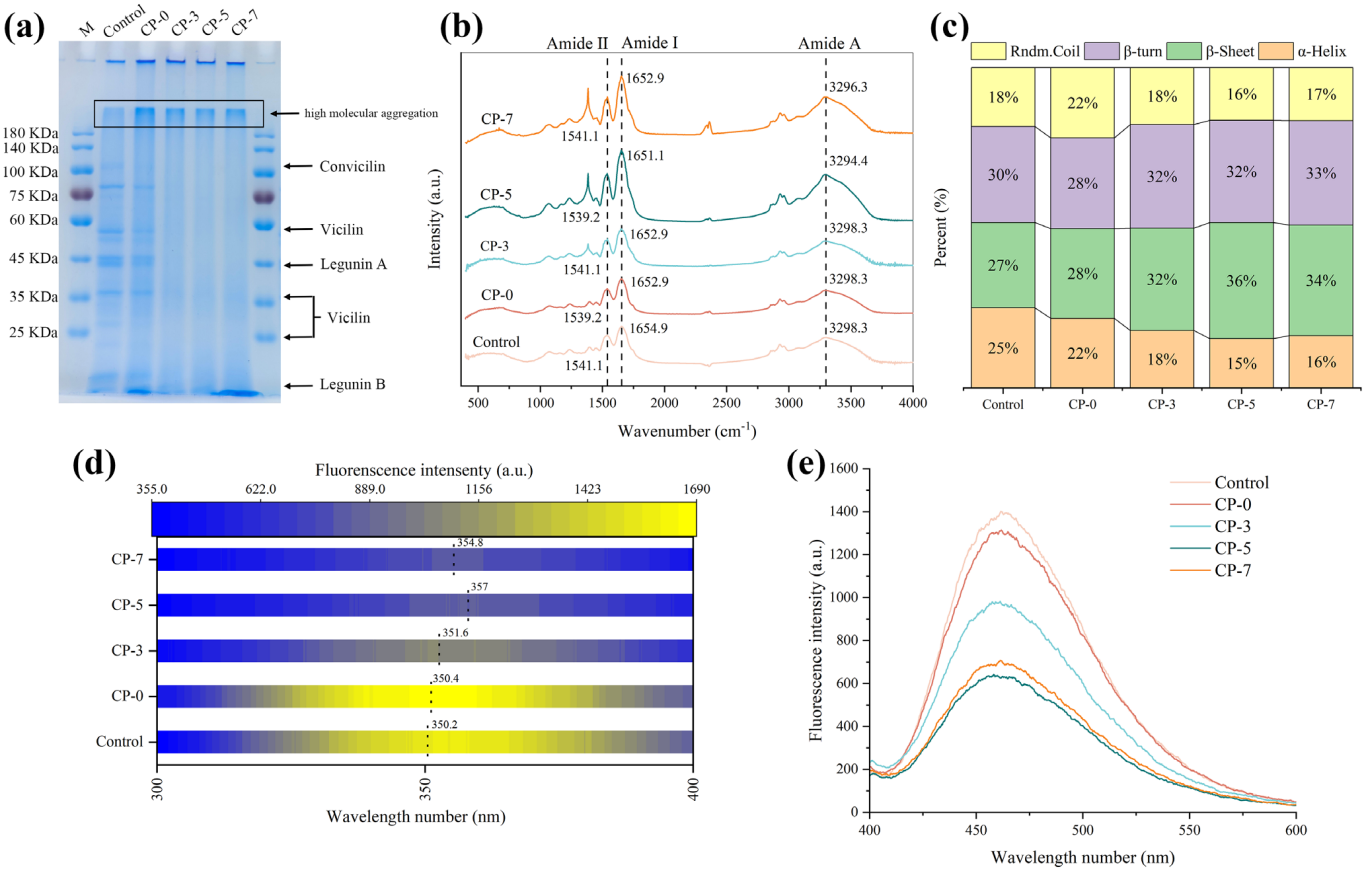
Change in SDS‐PAGE (a), FTIR spectra (b), the content of secondary structure (c), intrinsic fluorescence intensity (d), surface hydrophobicity (e) of PPI amyloid fibrils prepared by CP treated PPI for 0, 3, 5, and 7 min, respectively, with a fibrillation time of 10 h.

Food protein amyloid fibrils are fibrous protein structures formed through the self‐assembly of proteins or peptides. Typically, native proteins undergo conformational and structural transitions before assembling into amyloid fibrils during the fibrillation process. Characteristic structural changes in proteins during this process include unfolding their globular structure, hydrolyzing, or cleaving the backbone of the polypeptide chain, releasing the “β‐strands” building blocks structure, and assembling these blocks into fibrils (Xu, Zhou, et al. 2023). The protein pattern of CP‐treated PPI after 10 h of fibrillation was analyzed using reducing SDS‐PAGE. As shown in Figure 2a, the bands corresponding to 7S (convicilin ~67 kDa and vicilin ~48 kDa) and 11S (acidic subunit ~39 kDa and basic subunit ~22 kDa) proteins in samples treated with CP for 3, 5, and 7 min almost completely disappeared, while low molecular weight peptides and high molecular weight aggregates (> 180 kDa) were observed. For CP‐0, no significant changes in band patterns were observed, except for the appearance of high molecular weight aggregates compared with the native protein. These results suggest that cleavage of the polypeptide backbone in PPI occurs during the fibrillation process, likely induced by the active particles generated during CP treatment (Liu et al. 2025).

To quantify the changes in the secondary structure of PPI during the fibrillation process, FTIR analysis was performed. As illustrated in Figure 2b, the characteristic bands of PPI amyloid fibrils are the Amide I band (1700–1600 cm^−1^), Amide II band (1575–1480 cm^−1^), and Amide A band (3200–3400 cm^−1^), which are associated with C=O stretching vibrations, the coupling of C‐N stretching vibrations and N‐H bending vibrations, and the stretching vibration of N‐H bonds, respectively (Zhou et al. 2022). The calculated secondary structure content is shown in Figure 2c. For native PPI, the constituent of secondary structure is 25% α‐helix, 27% β‐sheet, 30% β‐turn, and 18% random coil. The α‐helix and random coil structures of PPI gradually decreased from 22% to 16% and from 22% to 17%, respectively, as CP exposure time increased. Conversely, the β‐sheet and β‐turn structures increased from 28% to 34% and from 28% to 33%, respectively. These FTIR findings were supported by the ThT fluorescence results, reflecting that the β‐sheet structure serves as the primary building unit and is self‐assembled and stacked into more continuous β‐sheet structures during the fibrillation process. Similar findings were reported for fibrillar structures in hemp seed protein (Kutzli et al. 2023) and oat globulin (Xu et al. 2024a).

Change in the intrinsic fluorescence intensity of CP‐treated PPI is present in Figure 2d. Upon heating, a significant decrease in maximum fluorescence intensity (FI_max_) and a red shift in the wavelength of maximum fluorescence intensity (*λ*_max_) were observed. The *λ*_max_ of PPI gradually shifted from 350.2 nm (Control) to 357 nm (CP‐5), and then declined to 354.8 nm for CP‐7. These results implied that structural unfolding of the PPI molecule during the fibrillation incubation resulted in exposing aromatic amino acid residues, previously buried in its globular structure, to a more polar aqueous environment. This increased microenvironmental polarity led to fluorescence quenching of PPI and red shift in *λ*_max_ (Tong et al. 2022). Similar findings were reported by Xu et al. (2024), who observed a gradual decrease in the maximum fluorescence intensity of oat globulin accompanied by a significant red shift in *λ*_max_ during a 24‐h fibrillation incubation.

Surface hydrophobicity indicates the extent to which hydrophobic sites are exposed on the protein surface. Notably, it is widely accepted that hydrophobic forces serve as the primary driving impetus for the assembly and stacking of building blocks (cross β‐sheets), leading to the formation of an ordered fibrous structure (Ouyang et al. 2025). As shown in Figure 2e, after 10 h of fibrillation incubation, the surface hydrophobicity of PPI was gradually reduced with CP treatment time of up to 5 min and slightly increased with further extended CP treatment time to 7 min. These results implied that cross β‐sheet building blocks self‐assembled and stacked into an ordered fibrous structure through hydrophobic interactions, thereby masking exposed PPI hydrophobic groups and reducing surface hydrophobicity. Taken together, these results align with the fibrillation of arachin (Yang, Wang, et al. 2024), oat globulin (Xu, Tang, Wang, Xie, and Xu, et al. 2024), and pea globulin (Liu, Yang, et al. 2024a), demonstrating that PPI amyloid fibrils were successfully promoted by CP treatment alone in combination with heat treatment. From the results of ThT and TME analysis, the PPI amyloid fibrils generated by treating with CP for 5 min (CP‐5) exhibited the most favorable performance. Therefore, amyloid fibrils formed by CP‐5 with different heating times (2, 4, 8, and 12 h) were selected for subsequent studies on HIPE stabilization.

### 3.2 Properties of HIPEs Stabilized by CP‐Induced PPI Amyloid Fibrils

#### 3.2.1 Characterization of HIPEs

**FIGURE 3:**
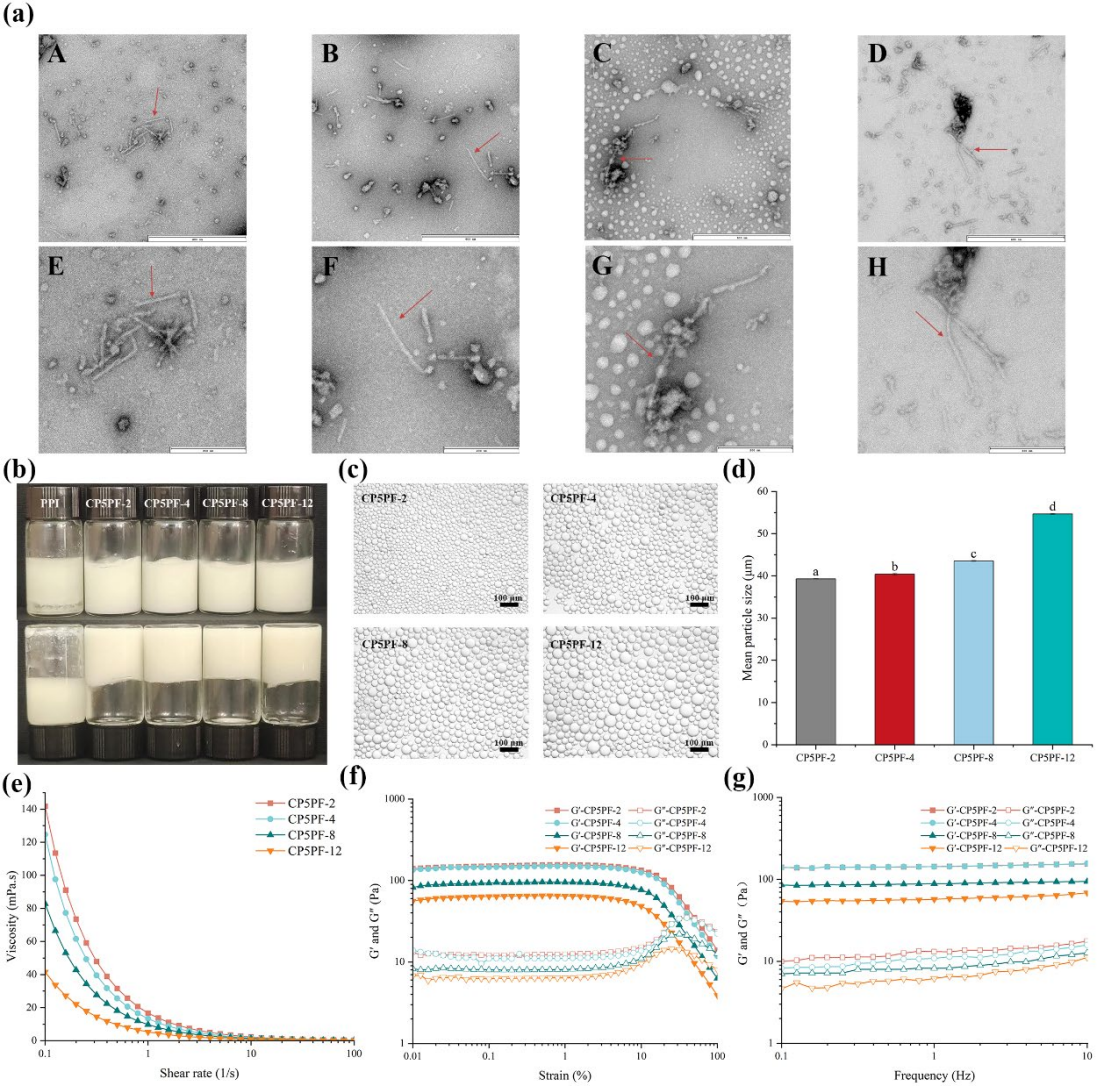
(a) TEM analysis of PPI amyloid fibrils prepared by CP‐treated PPI for 5 min with different fibrillation times (2, 4, 8, and 12 h). (A, E), (B, F), (C, G), (D, H) were CP‐treated PPI for 5 min with fibrillation times of 2, 4, 8, and 12 h, respectively. The scale bar of (A–D) is 600 nm, and the scale bar of (F–I) is 200 nm. (b) Visual appearance of HIPEs; (c) optical micrographs of HIPEs; (f) average oil droplet size of HIPEs; (e) apparent viscosity of HIPEs; (f) strain sweep of HIPEs at a frequency of 1.0 Hz; (f) frequency sweep of HIPEs at a constant strain of 0.03%. The amyloid fibrils by CP‐treated PPI for 5 min with different fibrillation times (2, 4, 8, and 12 h) were noted as CP5PF‐2, CP5PF‐4, CP5PF‐8, and CP5PF‐12, respectively.

The dynamic change in morphology of PPI amyloid fibrils formed by CP‐5 at different fibrillation times (2, 4, 8, 12 h) was illustrated by TEM analysis (Figure 3a). Compared with the control, the spherical morphology of native PPI disappeared (Figure 1b), replaced by short rod‐like fibrils with an average length of 132.21 nm, implying that building blocks (β‐sheet) underwent ordered self‐assembly into protein fibrils after 2 h of fibrillation incubation. Interestingly, it was displayed that the fibrils elongated as incubation time was prolonged from 2 to 12 h, with the length of PPI fibrils consistently increasing from 132.21 nm (2 h) to 177.92 nm (4 h), 221.19 nm (8 h), and 306.66 nm (12 h), respectively. The properties of the HIPEs prepared by the amyloid fibrils of CP‐5‐treated PPI (CP5PF) for different fibrillation times (2, 4, 8, 12 h) were shown in Figure 3b. Results indicated that the HIPEs were effectively stabilized by CP5PF, exhibiting a gel‐like structure capable of adhering to the bottom of an inverted container. In contrast, apparent oil release and phase separation phenomena followed in the HIPEs stabilized by native PPI, with the sample dripping from the inverted bottle. The morphology of oil droplets in HIPEs was captured by optical microscopy (Figure 3c), showing that droplet size significantly increased as the incubation time was extended from 2 to 12 h. These results were in line with the dynamic particle size distribution analysis (Figure 3d), where the average droplet size of the HIPEs stabilized by CP5PF gradually increased from 39.3 μm (2 h) to 54.6 μm (12 h). This indicated that the stability of HIPEs prepared by CP5PF was directly related to the length of PPI amyloid fibrils. As the fibrillation time prolonged, the length of PPI amyloid fibrils grew, as confirmed by TEM analysis (Figure 3a), while the stability of HIPEs deteriorated. This phenomenon may be attributed to the reduced mobility of longer PPI amyloid fibrils, which adsorb poorly onto oil droplet interfaces and form a less compact interfacial layer than shorter fibrils (Blijdenstein et al. 2004). Similarly, Liu et al. (2020) reported that nanofibrils derived from glycated whey protein isolate with reduced fibril length formed a thicker layer on oil droplets and produced more stable emulsions than conventional long, rigid amyloid fibrils of whey protein isolate.

**FIGURE 4:**
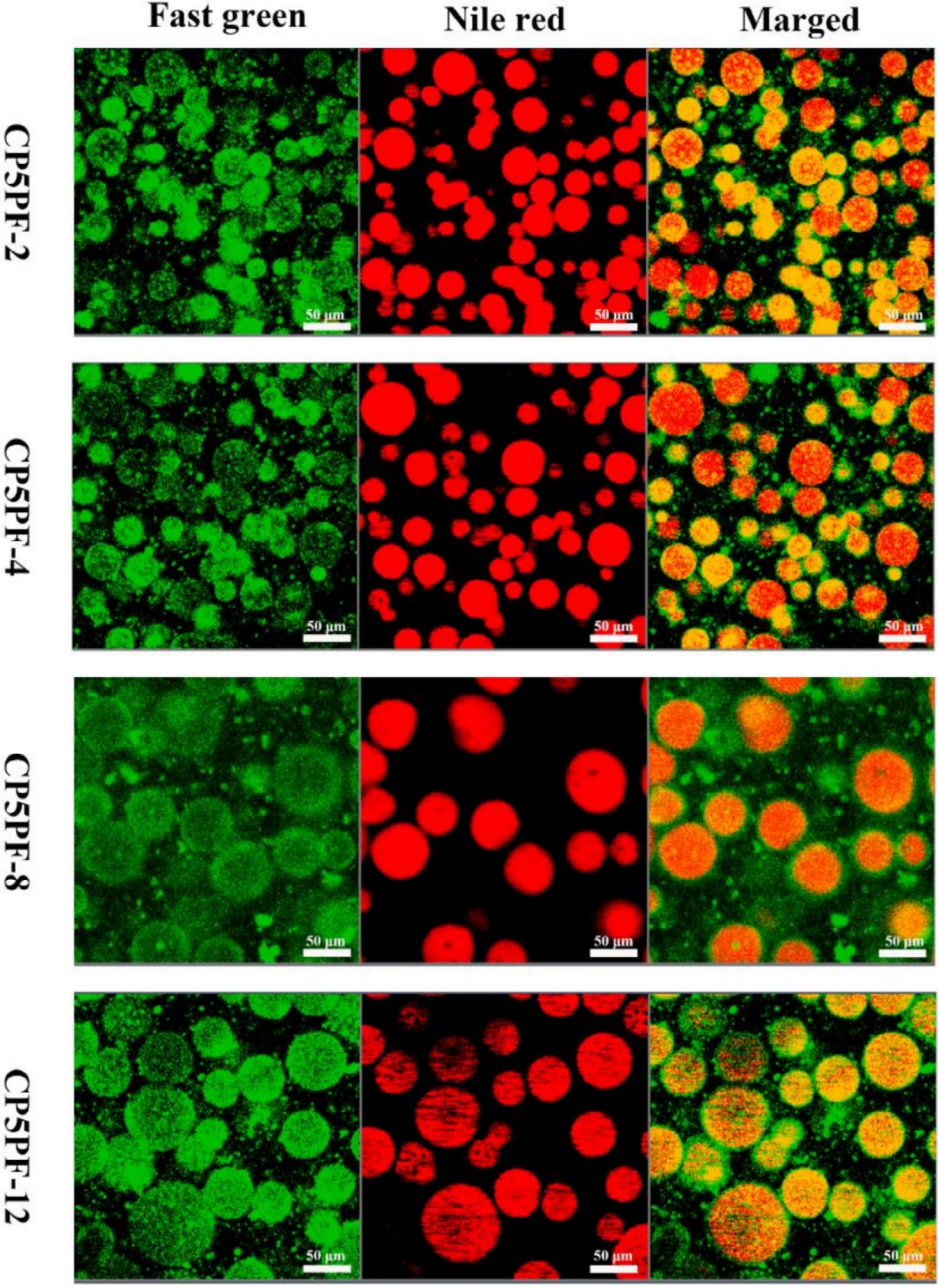
CLSM micrographs of HIPEs stabilized by PPI amyloid fibrils prepared by CP‐treated PPI for 5 min with different fibrillation time (2, 4, 8, 12 h). The oil phase was stained with Nile Red, and the dispersed phase was stained with Fast Green.

The morphology and the microstructure of the oil droplets in HIPEs stabilized by CP5PF were further illustrated by CLSM. In Figure 4, the oil phase appeared green, while the protein was colored red. All HIPE samples showed a typical oil‐in‐water (O/W) emulsion, with protein emulsifiers effectively adsorbed at the oil–water interface. Additionally, the oil droplet size of the HIPEs increased with prolonged incubation time, consistent with particle size and optical microscopy analysis (Figure 3c,d). A distinct green ring was observed around the red oil droplets, indicating that the amyloid fibrils were predominantly adsorbed at the interface between the oil and water phases. The amyloid fibrils encased the oil droplets, forming a protective film that prevented droplet aggregation by generating electrostatic and spatial repulsive forces, thereby enhancing the stability of the HIPEs (Zhao et al. 2024).

#### 3.2.2 Rheological Properties of HIPEs

Rheological properties analysis was employed to assess the variation in the microstructure of HIPEs stabilized by CP5PF with different fibrillation times. The apparent viscosity of HIPEs stabilized by CP5PF with varying incubation times during the fibrillation process was depicted in Figure 3d. The apparent viscosity of all HIPEs consistently decreased with increasing shear rate, exhibiting the typical shear‐thinning characteristic of non‐Newtonian pseudoplastic fluids (Xu, Ma, et al. 2023). This occurrence may be ascribed to the progressive disruption of the network structure formed by PPI amyloid fibrils on the oil droplet surface as shear rates increased, resulting in a reduction in the apparent viscosity of HIPEs under high shear rates (Xu, Ma, et al. 2023). On the other hand, the apparent viscosity of HIPEs gradually decreased with prolonged incubation time. This could be due to a stronger spatial barrier formed by shorter amyloid fibrils of PPI, which effectively prevent droplet flocculation and emulsion stratification, thereby improving the stability of HIPEs (Blijdenstein et al. 2004). Specifically, shorter fibrils formed during 2 or 4 h heat treatment times efficiently adsorb onto the oil droplet surface, lowering interfacial tension and facilitating the formation of small and homogeneous oil droplets. This results in an additional robust network structure, which hinders the oil droplet aggregation under firm shear conditions and thus enhances the viscosity.

Strain sweep analysis is crucial for assessing the viscoelasticity of emulsions. It effectively reveals the relationship between microstructures and macroscopic properties of HIPEs, reflected by the storage modulus (G′, elasticity) and loss modulus (G′′, viscosity) (Zhao et al. 2021). The strain dependence of G′ and G′′ in HIPEs stabilized by CP5PF at different fibrillation process times is displayed in Figure 3e, showing that all CP5PF‐stabilized HIPEs exhibited a linear viscoelastic region (LVR) in the lower strain amplitude range (> 10%). Generally, the wider the LVR, the better the resistance to deformation (Li et al. 2020). CP5PF‐stabilized HIPEs displayed typical solid‐like characteristics with G′ > G′′ (Liu, Li, et al. 2021). A gradual decrease in G′ and G′′ values was observed with increasing fibrillation time. These results were consistent with apparent viscosity analysis (Figure 3e), implying that shorter amyloid fibrils (formed at 2 and 4 h fibrillation times) adsorbed more effectively onto oil droplet surfaces, resulting in a compact interfacial structure and improved gel strength. Similar trends were observed in frequency sweep tests (0.03% strain), where G′ and G′′ lines were parallel with slight increases as frequency rose, showing an order of CP5PF‐2 > CP5PF‐4 > CP5PF‐8 > CP5PF‐12. Those results indicated that compared with the different morphologies of PPI fibrils induced by CP treated for 5 min with different fibrillation time durations fundamentally contribute to the improved stability of HIPEs, particularly in terms of average lengths. Shorter fibrils (CP5PF‐2 and CP5PF‐4) exhibit better mobility, enabling more efficient adsorption at the oil–water interface compared to longer fibrils (CP5PF‐8 and CP5PF‐12). The more flexible and anisotropic properties of the short rod‐like fibrils make them more effective at forming dense and elastic interfacial layers, thereby preventing droplet flocculation, coalescence, and improving overall emulsion integrity (Liu et al. 2020; Liu, Chen, et al. 2024; Liu, Tang, et al. 2024). To summarize, PPI amyloid fibrils promoted by CP treatment exhibited a strong capacity for stabilizing HIPE, and their ability to stabilize HIPE was dependent on fibril length. To broaden its application, the thermal, ionic, and storage stability of CP5PF‐stabilized HIPEs were further investigated.

### 3.3 Thermal, Ionic, and Storage Stability of HIPEs Stabilized by CP‐Induced PPI Amyloid Fibrils

#### 3.3.1 Thermal Stability of HIPEs

**FIGURE 5:**
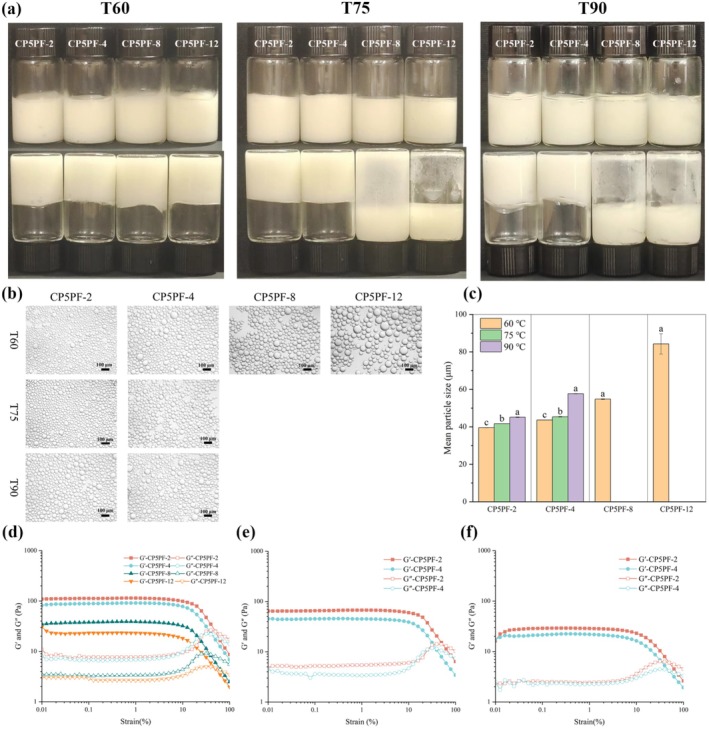
Thermal stability (60°C, 75°C, and 90°C) of HIPEs stabilized by PPI amyloid fibrils prepared by CP treated PPI for 5 min with different fibrillation times (2, 4, 8, 12 h): (a) Visual appearance; (b) Optical micrographs; (c) Average oil droplet size (c); (d–f) Strain sweep of HIPEs at a frequency of 1.0 Hz: (d) 60°C, (e) 75°C, (f) 90°C.

Heating is a common sterilization procedure in food processing to prevent bacterial contamination and extend food shelf life. The thermal stability of HIPEs stabilized by CP5PF was evaluated at 60°C, 75°C, and 90°C. The visual appearance of the HIPEs indicated that all CP5PF‐stabilized HIPEs exhibited good stability across all thermal treatment temperatures, except for HIPEs prepared with CP5PF‐8 and CP5PF‐12, where no gel‐like emulsion was formed at 75°C and 90°C (Figure 5a). Results from optical microscopy and dynamic particle size distribution analysis displayed that the particle size of oil droplets of CP5PF‐fabricated HIPEs significantly increased as the heating temperature rose from 60°C to 90°C (Figure 5b,c).

The detailed information in the microstructure of HIPEs and the interaction between droplets was further elucidated by strain dependence sweep of rheological analysis, which indicated that heat treatment has a significant influence on the stability of all CP5PF‐fabricated HIPEs. It clearly displayed a significant decline in G′ and G′′ values across all samples at 60°C, 75°C, and 90°C. The extent of decline intensified with increasing temperature. However, compared with HIPEs stabilized by CP5PF‐8 and CP5PF‐12, the HIPEs prepared by CP5PF‐2 and CP5PF‐4 displayed greater stability at all temperatures (Figure 5d–f). These findings confirmed that PPI amyloid fibrils (CP5PF‐2 and CP5PF‐4) with shorter fibrils lengths efficiently adsorbed at the oil droplet interface, forming a compact network structure that is more resistant to heat treatment (Tan et al. 2024).

#### 3.3.2 Ionic Stability of HIPEs

**FIGURE 6:**
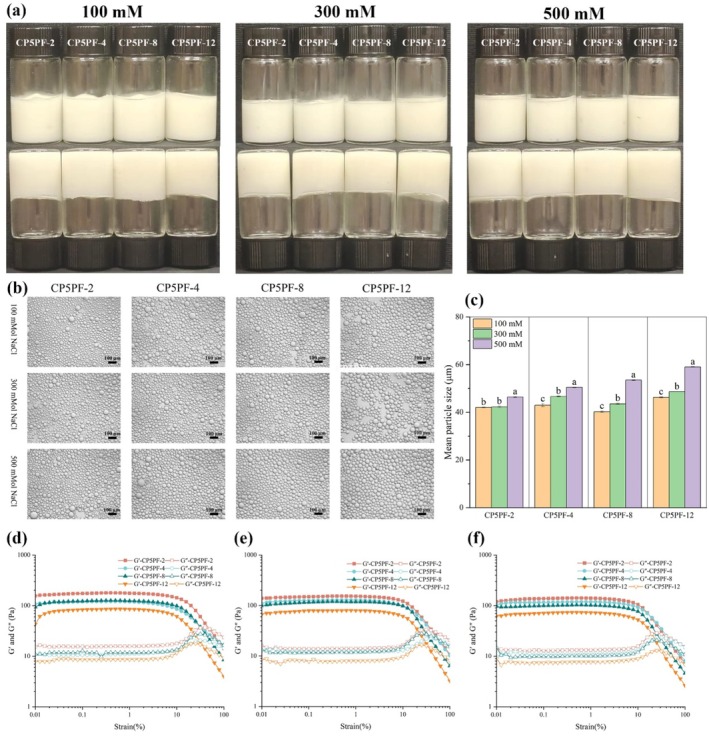
Ionic stability (100, 300, and 500 mM NaCl) of HIPEs stabilized by PPI amyloid fibrils prepared by CP treated PPI for 5 min with different fibrillation times (2, 4, 8, 12 h): (a) Visual appearance, (b) optical micrographs, (c) average oil droplet size, (d–f) strain sweep of HIPEs at a frequency of 1.0 Hz: (d) 100 mM, (e) 200 mM, (f) 300 mM.

In food processing, the addition of NaCl inhibits the growth of bacteria and molds, extending the shelf life of food. The effect of ionic strength (100–500 mM NaCl) on the appearance, micromorphology, and oil droplet size of HIPEs prepared by CP5PF at different incubation times was depicted in Figure 6. Results indicated that all HIPE samples did not undergo phase separation and maintained a gel‐like appearance (Figure 6a). Additionally, the oil droplets of HIPEs exhibited uniform and homogeneous particle sizes across all ionic strengths, tightly aligning and adhering to each other (Figure 6b). These findings were consistent with dynamic particle size analysis results (Figure 6c). Rheological analysis (Figure 6d–f) showed that G′ values remained higher than G′′ values at all ionic strengths, demonstrating typical colloidal behavior and excellent ionic stability. Compared to samples without added ions (Figure 3f), ionic strength had little effect on the rheological properties of HIPEs stabilized by CP5PF, suggesting that the presence of charged ions enhanced oil droplet binding and emulsion hardness. This phenomenon may be attributed to electrostatic repulsion between PPI amyloid fibrils being shielded by NaCl ions, promoting particle diffusion and adsorption at the oil–water interface, thereby increasing the stability of CP5PF fabricated HIPEs (Qiu et al. 2023). Similar findings were reported by He et al. (2024), who observed enhanced stability of walnut protein amyloid fibril‐fabricated HIPEs at high ionic strength (500 mM NaCl).

#### 3.3.3 Storage Stability of HIPEs

**FIGURE 7:**
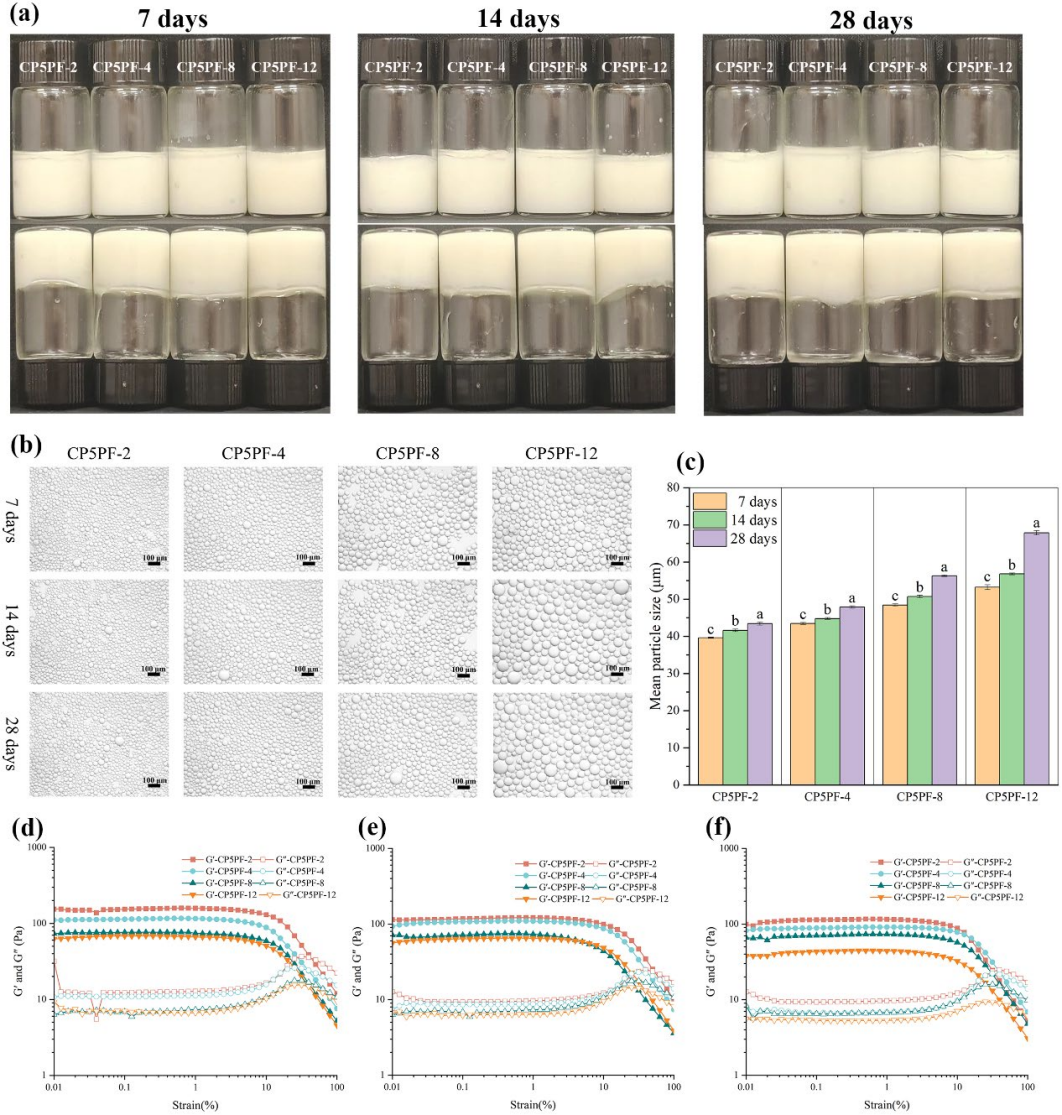
Storage stability (7, 14, and 28 days) of HIPEs stabilized by PPI amyloid fibrils prepared by CP treated PPI for 5 min with different fibrillation times (2, 4, 8, 12 h). (a) visual appearance, (b) optical micrographs, (c) average oil droplet size, (d–f) strain sweep of HIPEs at a frequency of 1.0 Hz: (d) 7 days, (e) 14 days, (f) 28 days.

The storage stability of CP5PF‐stabilized HIPEs over a 28‐day period is shown in Figure 7. No significant differences in the appearance of HIPEs were observed during the storage period; no oil precipitation or emulsion delamination occurred, and the emulsions remained highly self‐supporting after 28 days of inverted placement (Figure 7a). Micromorphology and dynamic particle size analysis revealed a gradual increase in oil droplet size with extended storage time. However, compared with HIPEs stabilized by CP5PF‐8 and CP5PF‐12, the increase in oil droplet size was smaller in HIPEs stabilized by CP5PF‐2 and CP5PF‐4 (Figure 7b,c). Strain sweep rheological analysis yielded similar results, showing slight declines in G′ and G″ values for all HIPEs over time. However, the decline was significantly larger for CP5PF‐8 and CP5PF‐12 than for CP5PF‐2 and CP5PF‐4 (Figure 7d–f). Overall, these findings demonstrate that CP5PF‐stabilized HIPEs exhibited excellent storage stability, with shorter‐length PPI amyloid fibrils (CP5PF‐2 and CP5PF‐4) outperforming longer fibrils.

## 4 Conclusions

The formation of PPI amyloid fibrils induced by CP treatment merged exclusively with the heat treatment was demonstrated. As compared to native PPI, the stability of HIPEs stabilized by the CP‐induced PPI amyloid fibrils was significantly enhanced. Results from investigations into the properties of HIPEs showed that the stability of HIPEs was directly related to the length of rod‐like PPI amyloid fibrils. This may be ascribed to the statement that longer PPI amyloid fibrils are less mobile, adsorb less efficiently onto the interface of oil droplets, and form less compacted interfacial layers compared to shorter fibrils. In conclusion, CP offers a promising alternative to conventional, time‐consuming acidic‐heat treatments to induce PPI amyloid fibril formation and improve their capacity to stabilize HIPEs. Although the ability of CP treatment to facilitate protein amyloid fibril formation was confirmed, further studies on regulating the shapes and characteristics of protein amyloids and their enhanced functionality in practical applications should be undertaken.

## Author Contributions

**Jing Wang:** data curation (equal), writing – original draft (equal). **Jun‐Xiang Liu:** data curation (equal), writing – review and editing (equal). **Xiu‐Bin Liu:** writing – review and editing (equal). **Najla AlMasoud:** writing – review and editing (equal). **Abderrahmane Aït‐Kaddour:** funding acquisition (equal), writing – review and editing (equal). **Rana Muhammad Aadil:** funding acquisition (equal), investigation (equal), supervision (equal), writing – review and editing (equal). **Zhi‐Wei Liu:** data curation (equal), investigation (equal), methodology (equal), resources (equal), software (equal), supervision (equal), writing – original draft (equal), writing – review and editing (equal).

## Conflicts of Interest

The authors declare no conflicts of interest.

## Data Availability

Data available on request due to privacy/ethical restrictions.
